# Protocol for quantitative nuclear magnetic resonance for deciphering electrolyte decomposition reactions in anode-free batteries

**DOI:** 10.1016/j.xpro.2022.101867

**Published:** 2022-11-19

**Authors:** Ming-Yue Zhou, Xiao-Qing Ding, Li-Peng Hou, Jin Xie, Bo-Quan Li, Jia-Qi Huang, Xue-Qiang Zhang, Qiang Zhang

**Affiliations:** 1 Beijing Key Laboratory of Green Chemical Reaction Engineering and Technology, Department of Chemical Engineering, Tsinghua University, Beijing 100084, China; 2 Advanced Research Institute for Multidisciplinary Science, Beijing Institute of Technology, Beijing 100081, China; 3 School of Materials Science and Engineering, Beijing Institute of Technology, Beijing 100081, China

**Keywords:** NMR, Energy, Chemistry, Material sciences

## Abstract

In this protocol, we describe the quantification of electrolytes using nuclear magnetic resonance. We detail the steps involved for battery cycling, sample preparation, instrument operation, and data analysis. The protocol can be used to quantify electrolyte decomposition reactions and the apparent electron transfer numbers of different electrolyte components. The protocol is optimized for lithium-based anode-free batteries but can also be applied to other rechargeable batteries.

For complete details on the use and execution of this protocol, please refer to Zhou et al. (2022).^1^

## Highlights

• A qNMR approach for electrolyte quantification after cycling and decomposition
• Optimized sample preparation, instrument operation, and data analysis for precision
• Anode-free configuration and inactive Li titration for determination of capacity
• Protocol for acquiring apparent electron transfer numbers of electrolyte decomposition

## Abstract (editor highlights)

Publisher’s note: Undertaking any experimental protocol requires adherence to local institutional guidelines for laboratory safety and ethics.

## Graphical abstract

**Figure:**
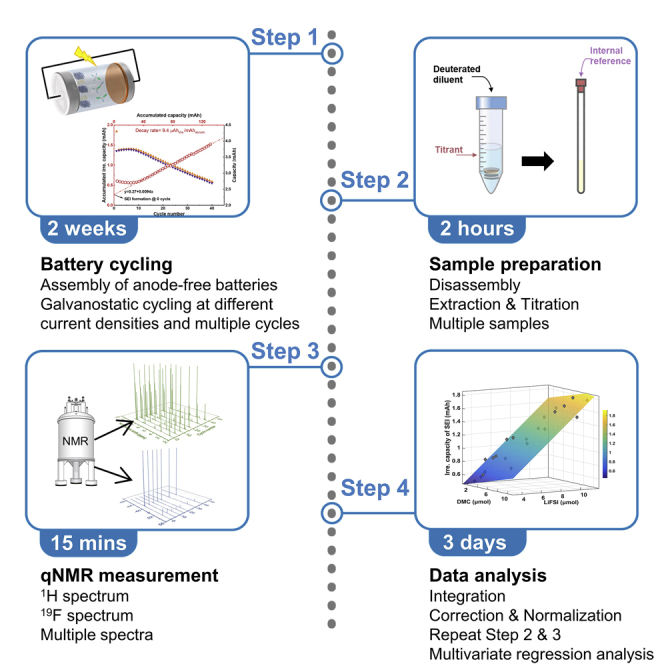

## Before you begin

In non-aqueous secondary batteries, the electrolyte decomposition reactions at the anode/electrolyte interface are considered to be the most important and complex phenomenon.^2^ Electrolyte decomposition reactions lead to irreversible capacity decay and the formation of solid electrolyte interphase (SEI), which dictates the performance and stability of the anode. However, because of the complexity of simultaneous reactions of multiple components of the electrolyte and the interference of inevitably formed inactive lithium,^3^ it is hard to accurately measure the change of different electrolyte components and assign the corresponding electron consumption.

In the battery community, qNMR has been proven to be a powerful tool to investigate the electrolyte composition and its evolution upon cycling in various systems.^4^^,^^5^^,^^6^^,^^7^^,^^8^ Herein, the procedure of qNMR for electrolyte quantification and the recently developed electron transfer numbers (ETNs) fitting method are documented and elucidated.

Normally, the electrolyte should be prepared beforehand, though the commercialized electrolyte with known components can be used as well. The deuterated diluent that contains titrant should also be prepared. Moreover, the software for quantitative NMR (qNMR) data processing and multivariate regression analysis is also necessary, which are JEOL Delta and MATLAB in this protocol. As for the battery configuration, the anode-free battery, namely a battery without lithium metal, should be used to reflect the total irreversible capacity loss of the anode.^9^ Besides, multiple samples at different C-rates are required for the subsequent apparent ETN fitting. The protocol below describes the specific steps for using coin cells with dimethyl carbonate (DMC)-based high-concentration electrolyte (HCE). We have also used this protocol in anode-free pouch cells and with other types of electrolyte.^1^

### Electrolyte preparation

**Timing: 30 min**

1. Determine the composition and molar ratio of electrolyte components [10 min].
  a. For this particular protocol, the composition of electrolyte is set up to be lithium bis(fluorosulfonyl)imide (LiFSI), DMC with a molar ratio of 1.0: 1.6.
  b. Other salts [e.g., lithium bis(trifluoromethane sulfonyl) imide (LiTFSI) and lithium hexafluorophosphate (LiPF_6_)] and solvents [e.g., 1,2-dimethoxyethane (DME) and ethylene carbonate (EC)] can be also used as long as the salt has fluorine atoms and the solvent has hydrogen atoms.
2. Calculate and weigh the electrolyte components [10 min].
  a. Normally, the volume of solvent is determined in the first place, as the base to calculate the other components. For instance, 0.5 mL DMC is generally sufficient for one batch of an experiment (20 sample cells).
  b. Weigh the salt by electronic balance and transfer the solvent by pipette into a sample tube in an Ar-filled glovebox.
  c. The molar ratio of the prepared electrolyte would usually deviate from the calculated value due to the evaporation of solvent caused by the exothermic dissolving process.
  d. For the high-concentration electrolyte in this protocol, the ratio of the as-prepared 0.5 mL electrolyte would deviate to ∼1.0:1.45.
  e. Adding ∼0.05 mL extra DMC can compensate for the loss in this particular protocol. Slowly adding the salt into the solvent or cooling the solvent may also eliminate the deviation.
3. Mix until the salt is dissolved by shaking the electrolyte at 25 degrees Celsius (by a vortex mixer or simply by hand) [10 min].
  a. The storage temperature for electrolytes is 25 degrees Celsius with a maximum storage time of < 1 month.

**CRITICAL:** The concentration of electrolytes may vary after storage. If possible, it is suggested to use electrolytes within 1 month after it was prepared.

### Battery materials preparation

**Timing: 30 min+24 h**

4. Determine the type and dimension of the battery [5 min].
  a. Stainless steel (SS) 2025-type coin cells with SS spacers of 0.5 mm thickness are used in this protocol.
5. Prepare the cathode, anode, separator, and aluminum spacer [25 min].
  a. Cut the cathode that is coated on aluminum foil into disks with diameters of 13 mm.
  b. Here, commercially available LiNi_0.5_Co_0.2_Mn_0.3_O_2_ (NCM523) is used. The ratio of active material, conductive additive, and binder is 96:2:2. The area capacity is 2.7 mAh cm^−2^.
    **CRITICAL:** Although the following results can be normalized by the measured capacity, the uniformity of cathodes is still critical for evaluating the consistency of results.
  c. Cut copper (Cu) foil into disks with diameters of 16 or 17 mm, which is used as the anode.
  d. Cut polyethylene (PE) separator into 19 mm disks.
  e. Cut aluminum (Al) foil into 19 mm disks, which is for the cathode.
6. Dry the above materials and transfer them into the glove box [24 h].

**CRITICAL:** Although the following results can be normalized by the measured capacity, the uniformity of cathodes is still critical for evaluating the consistency of results. Water impurities can deteriorate the battery performance through side reactions such as the reaction with lithium, the overcharge reaction with the cathode, and the hydrolysis reaction with electrolyte components (like LiPF_6_) to deteriorate the cathode. Those side reactions complicate the analysis and are out of the scope of this protocol. To ensure repeatable results, all materials should be properly dried. For the material mentioned in this particular protocol, vacuum drying for 24 h or placing in the glovebox (H_2_O < 0.1 ppm) for one week at 25 degrees Celsius is suggested.

### Diluent preparation

**Timing: 30 min**

7. Determine the composition of the extraction solution (diluent) [10 min].
  a. The diluent solvent for extraction solution should be deuterated, non-volatile, and readily miscible with electrolyte. Deuterated dimethyl sulfoxide (DMSO-d6) is chosen in this protocol.
  b. The titrant should be sufficiently reactive towards lithium metal (compared with the diluent and electrolyte) yet relatively unreactive towards other components. Maleic acid (MA) is chosen in this protocol.
  c. The relaxation enhancer should be paramagnetic and relatively soluble in the deuterated solvent. Chromium acetylacetonate [Cr(acac)_3_] is chosen in this protocol.

**CRITICAL:** The aim of using titrant is to illuminate the influence of remaining lithium metal. The aim of using the relaxation enhancer is to ensure the efficiency and accuracy of the qNMR measurement. For a detailed rationale of the choice of diluent, titrant, and relaxation enhancer, please refer to Zhou et al.^1^

8. Calculate and weigh the extraction solution [15 min].
  a. Normally, the volume of diluent solvent is determined in the first place, as the base to calculate the other components. For instance, 40 mL DMSO-d6 is generally sufficient for one batch (20 sample cells) of an experiment.
  b. Weigh titrant (MA) and relaxation enhancer [Cr(acac)_3_] into the container by an electronic balance in the glove box. Here, 100 mM of MA and 0.5 mM of Cr(acac)_3_ are used.
  c. Transfer diluent solvent (DMSO-d6) into the container by pipette in the glove box.
9. Mix until the solution is clear by shaking at 25 degrees Celsius (by a vortex mixer or simply by hand) [5 min].
  a. The storage temperature for extraction solution is 25 degrees Celsius with a maximum storage time of < 1 month.

**Table:** Composition of extraction solution

Ingredient	Selected reagent	Amount
Diluent solvent	DMSO-d6	40 mL
Titrant	Maleic acid	100 mM
Relaxation enhancer	Cr(acac)_3_	0.5 mM

### Installation of the software

**Timing: 1 h**

10. Install the JEOL Delta software [30 min].
  a. The JEOL Delta software can be downloaded from https://download.jeol.com.cn/download/delta-6.0.0-windows-x64-installer.zip. A permanent license key can be applied through https://nmrsupport.jeol.com/.
  b. Other software with similar functionality can also be used.
11. Install the MATLAB software [30 min].
  a. The MATLAB software can be downloaded from ww2.mathworks.cn/en/products/matlab.html. After registration, a free 30-day trial can be requested.
  b. For permanent usage, a license can be purchased for use by commercial or government organizations, degree-granting institutions, or individuals.
  c. Other software with similar functionality can also be used.

## Key resources table

**Table:** 

REAGENT or RESOURCE	SOURCE	IDENTIFIER
**Chemicals, peptides, and recombinant proteins**		

Deuterated dimethyl sulfoxide	Anhui Zesheng Technology Co., Ltd.	Cat#E090002
Maleic acid	Shanghai Macklin Biochemical Co., Ltd	Cat#M813428-25g
2,4-Dichlorobenzotrifluoride	Adamas Reagent Co., Ltd.	Cat#47952A
Chromium(III) acetylacetonate	Innochem (Beijing) Technology Co., Ltd.	Cat#A49115
Lithium bis(fluorosulfonyl)imide	Suzhou Duoduo Chemical Technology Co., Ltd.	Cat#9101701
Dimethyl carbonate	Suzhou Duoduo Chemical Technology Co., Ltd.	Cat#9200201
Dimethoxyethane	Suzhou Duoduo Chemical Technology Co., Ltd.	Cat#9200401
1,1,2,2-Tetrafluoroethyl-2,2,3,3-tetrafluoropropylether	Suzhou Duoduo Chemical Technology Co., Ltd.	Cat#9204101
LiNi_0.5_Co_0.2_Mn_0.3_O_2_ cathode	Guangdong Canrd New Energy Technology Co., Ltd.	Cat#SY1502
Polyethylene separator	Asahi Kasei Technosystem Co., Ltd.	
Copper foil	Hefei Kejing Materials Technology Co., Ltd.	

**Critical commercial assays**		

Battery tester	Neware	https://www.neware.com.cn/
NMR spectrometer JNM-ECZ400S	JEOL	https://www.jeol.co.jp/en/products/category_nmr.html
Hydraulic coin cell crimpers MSK-110	MTI Corporation	https://www.mtixtl.com/CompactHydraulicCrimpingMachineOneforAllButtonCells-MSK-110.aspx

**Software and algorithms**		

JEOL Delta v5.3.1	JEOL	https://nmrsupport.jeol.com/
MATLAB 2015a	MathWorks	https://www.mathworks.com/
Microsoft Excel 2020	Microsoft	https://www.microsoft.com/en-us/microsoft-365/excel
OriginPro 2021	OriginLab	https://www.originlab.com/

***Alternatives:*** This protocol has no special requirement on the source of reagents. The chemicals from other suppliers such as Sigma-Aldrich and Alfa Aesar can also be used*.* The use of electrode materials with different loading or type, separators with different porosity, and Cu current collectors with different types should not have an impact on the feasibility of this protocol as long as they do not change their inertness towards lithium and the extraction solution. Instruments and software with similar functions can also be used.

## Step-by-step method details

### Battery cycling

**Timing: ∼2 weeks (depending on the charging and discharging rates)**

Considering that the electrolyte decomposition reaction is self-limited, the battery cycling step aims to repeatedly form new interfaces and accumulate electrolyte changes, which ensures the reliability and repeatability of subsequent measurements. In anode-free batteries, as there is no excessive lithium at the anode, the capacity decay mainly reflects the loss of active lithium during the lithium plating/striping process at the anode side because the anode exhibits a much lower Coulombic efficiency than the cathode. Therefore, the accumulated irreversible capacity of the lithium plating/striping-type anode can be determined by the capacity change of the anode-free batteries.^9^ It should be noted that if an anode with higher Coulombic efficiency (but still less than the cathode) is used, the capacity retention and the testing time will be prolonged.

1. Assemble the Cu | NCM anode-free coin cells in an Ar-filled glovebox (Figure 1A) [20 min].
  **CRITICAL:** The consistency of batteries is important, which can be reflected by the battery performance i.e., capacity retention and voltage curve. It is suggested to carefully keep every condition identical, such as the assembly time and sealing pressure of each cell.
  a. Place an Al disk into the positive cell case.
  b. Place a cathode disk onto the Al disk.
  c. Add a controlled amount of electrolyte (10 μL) onto the cathode disk.
  d. Place a PE disk onto the electrolyte.
  e. Add a controlled amount of electrolyte (10 μL) onto the PE disk.
  f. Place a Cu disk onto the electrolyte.
  g. Place a SS spacer and spring onto the Cu disk.
  h. Place the negative cell case onto the positive cell case.
  i. Use a hydraulic coin cell crimper to seal the battery (750 psi).

**Figure 1:**
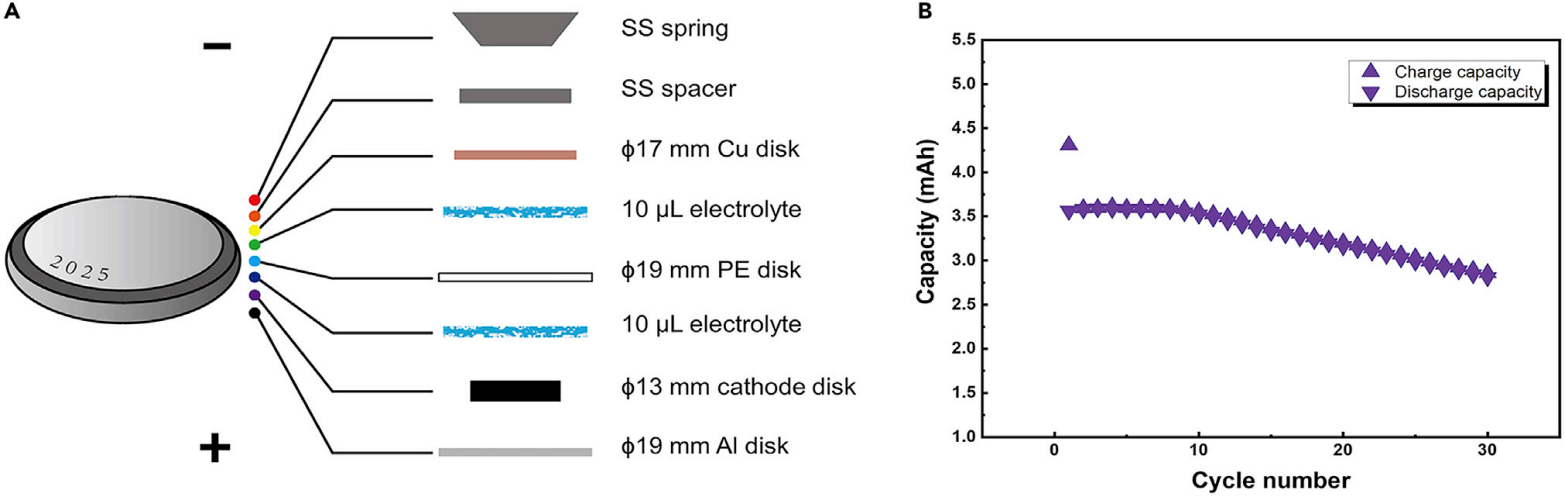
Battery assembly and cycling (A) The architecture of the anode-free cell. (B) Typical capacity retention data.
2. Use the battery tester to cycle the cells at constant currents [∼2 weeks (depending on the charging and discharging rates)].
  a. Rest 24 h before cycling.
  b. Leave 2 cells uncycled.
  c. Charge and discharge the other cells at C-rates of 0.2C, 0.4C, and 0.8C at constant current (CC) mode with 15 min resting step in between cycles.
  d. Set the cycle loop to be 10, 20, 30, and 40 cycles.
  e. Set the cutoff voltage range to 2.8–4.3 V.
  f. Set the temperature to 25°C.
3. Collect the cells and corresponding battery data (Figure 1B) [20 min].
  a. Record and plot the capacity retention data using OriginPro.

### Sample preparation

**Timing: 2 h**

The sample preparation step has two main targets. One is to achieve thorough extraction of the electrolyte, which is the basis for the correct qNMR calculation. Another one is to perform the pre-titration of the remaining Li metal, which provides quantitative information on the remaining Li metal and avoids the continuous consumption of electrolytes during preparation.

4. Use volatile solvent to wash and clean the die of the coin cell unsealing machine as well as the coin cell cases. Here, dimethoxyethane (DME) is chosen [10 min].
5. Use a pipette to transfer a certain volume (V_1_) of the extraction solution into containers with diameters larger than 20 mm. Here, 2 mL of diluent is used (Figure 2) [1 min].

**Figure 2:**
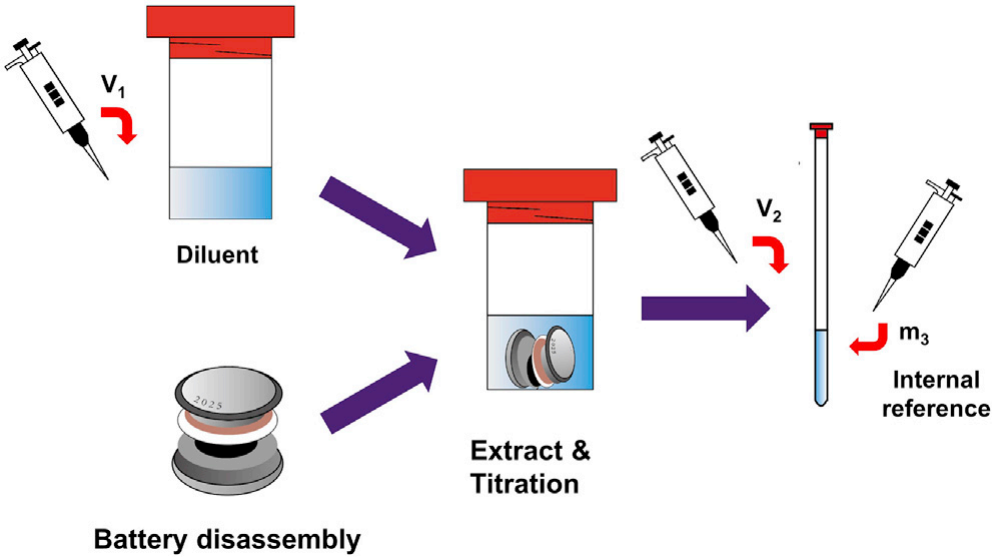
The schematic diagram of the sample preparation process
6. Use the hydraulic coin cell crimpers to disassemble the cycled cells in an Ar-filled glove box [1 min].
7. Transfer opened cells immediately into the container containing the extraction solution. Then close and seal the container [<3 s].
8. After ∼5 min for the inactive Li metal titration (by visually inspecting if there is blackish deposition on Cu foil), use an automatic shaker to shake the container for 40 min [∼45 min].

**CRITICAL:** The thorough and even mixing is important, which significantly influences the quality of qNMR results. It is suggested to manually and visually check the cell components during the shaking process, since some components like cell cases may occasionally be sucked on the bottom of the container.

9. Rest for 40 min to mix thoroughly and evenly [40 min].
10. Add a certain amount (m_3_) of internal reference (DCBF) into NMR tubes. Here, 20 μL of DCBF is used [10 min].
11. Transfer a certain volume (V_2_) of the mixed solution into NMR tubes. Here, 0.6 mL of the mixed solution is used [10 min].

### qNMR measurement

**Timing: 15 min (for step 12)**

The qNMR experiment measures the number of active nuclei in the samples, which can reflect the concentration of certain species.^10^

**Figure 3:**
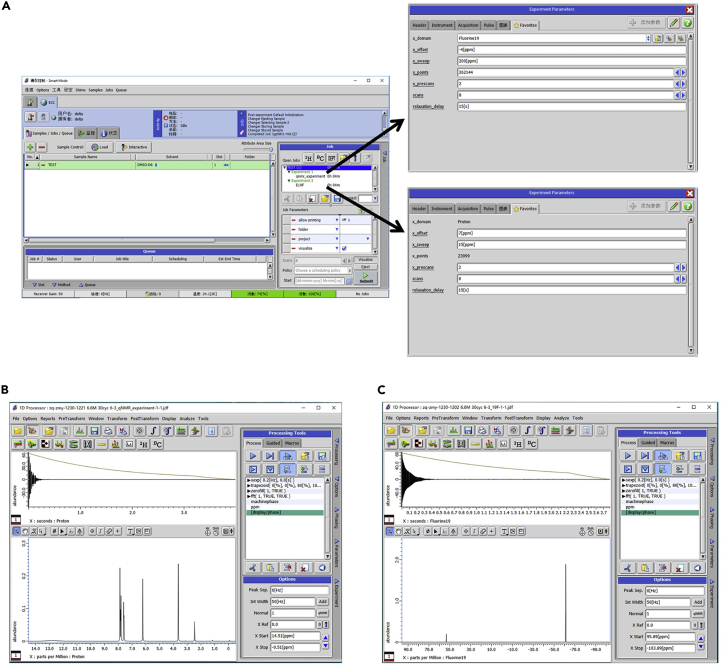
Software screenshots for qNMR measurements (A–C) (A) Settings of NMR parameters and acquired (B) ^1^H and (C) ^19^F spectra.

12. Load the NMR tubes into the NMR spectrometer [3 min].
13. Open the control console and set a pulse angle of 90° with a delay time (d1) of 15 s (Figure 3A) [30 s].

**CRITICAL:** The d1 should be 5 times larger than the slowest longitudinal relaxation time (T1) to ensure full relaxation between the pulses. A small amount of relaxation enhancer can significantly reduce the T1 and thus shorten the testing time.^11^ However, the optimal amount of relaxation enhancer may be varied in different samples. It is still suggested to measure the T1 before conducting a new experiment.

14. Set the transmitter offset at the center of signals [30 s].
15. Set the scan range to 120% of the interested signal range [30 s].
16. Set the scan number to 8, which can obtain a signal-to-noise ratio at least above 800 [30 s].
17. Run the NMR measurements to acquire the ^1^H and ^19^F spectra (Figure 3B) [10 min].

### Data analysis

**Timing: 3 days**

Through the data analysis step, the relative and absolute molar value of electrolyte components and the titrant can be determined, based on the known amount of internal reference.

18. Correct the chemical shift using Tetramethylsilane (TMS) and 2,4-Dichlorobenzotrifluoride (DCBF) as reference for ^1^H and ^19^F spectra, respectively, using JEOL Delta software [10 min].
19. Correct the phase and baseline of spectra using JEOL Delta software [10 min].
20. Extract and integrate the peak area JEOL Delta software (Figure 4A) [20 min].
  a. Use the automatic peak selection and integration method provided by JEOL Delta software. An example of the raw integral data can be found in Table 1.
    ***Optional:*** Special notice is that sometimes the interested peak may interfere with others, which requires further peak fitting using the Voigt function.
  b. Keep the same integration width for each signal. An example of the integration results can be found in Table 2.

**Table 1:** Example of raw data measured by NMR

Sample ID	Cycle number	*I*(DMC)^1^^H^ [*I*(A)^1H^]	*I*(FSI)^19^^F^	*I*(DMSO)^1^^H^	*I*(MA)^1^^H^	*I*(DCBF)^X^ [*I*(IR)^X^]
0	0	58.06	11.56	11.55	26.74	100
1	30	54.76	10.03	11.32	25.2	100

**Table 2:** Example of calculated results for ETN fitting

Sample ID	*n*(DMC)/*n*(FSI)	*n*(DMC) [μmol]	*n*(FSI) [μmol]	*n*(MA) [μmol]	*Δn*(DMC) [μmol]	*Δn*(FSI) [μmol]	*Δn*(MA) [μmol]
0	1.67	148.05	88.43	204.56	–	–	–
1	1.82	142.48	78.29	196.70	5.58	10.15	7.86
  c. At least five data points above the half-height of each peak are required to ensure integration accuracy.

**Figure 4:**
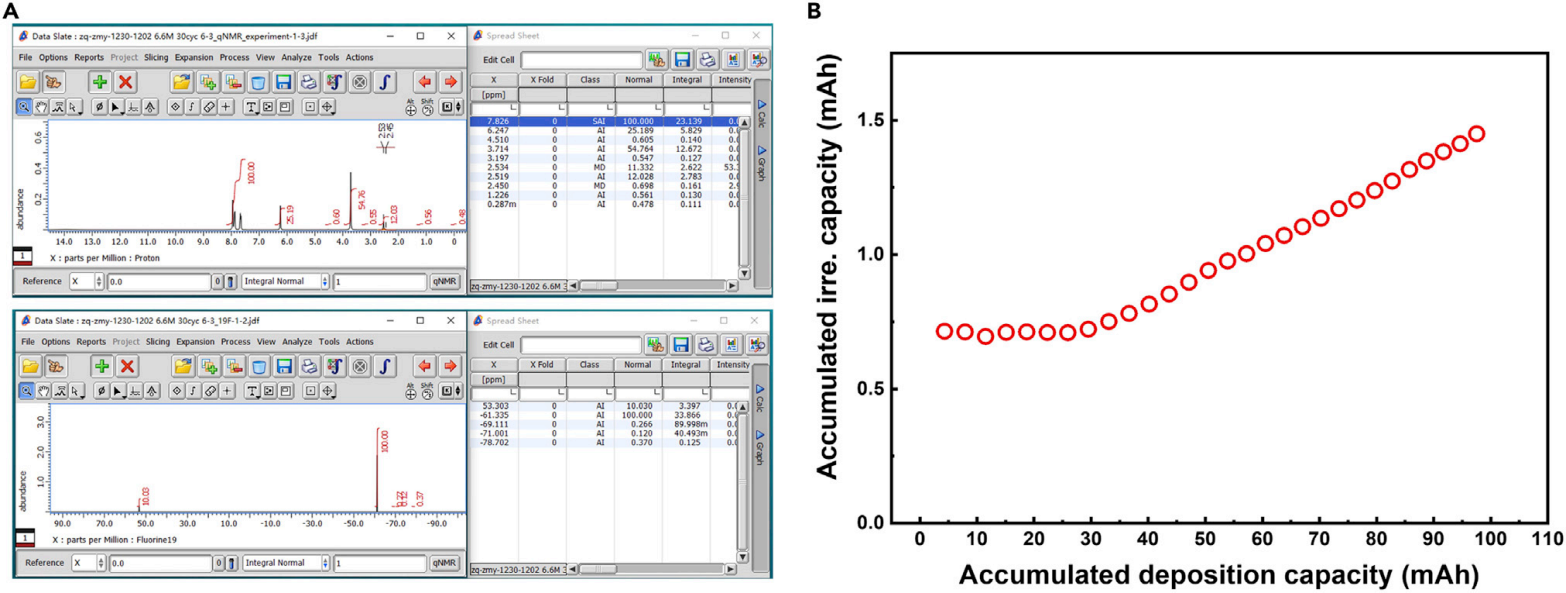
NMR integration and irreversible capacity calculation (A) Integration of 1H (upper) and 19F (lower) spectra using Delta software. (B) A typical irreversible capacity calculated based on the anode-free coin cell data.
21. Calculate the irreversible capacity of anode-free coin cells based on the capacity retention data and the following equations (Figure 4B) [20 min]:
  a. The n^th^ Accumulated deposition capacity= $\sum_{1}^{n}charge\;capacity$
  b. The n^th^ Accumulated irre.capacity=1^st^ charge capacity − the n^th^ discharge capacity.
22. Calculate the relative content of different electrolyte components using the equation [5 min]:

$$\frac{n{\left(\text{A}\right)}_{i}}{n{\left(Z\right)}_{i}}=\left(\frac{I{\left(A\right)}_{i}^{1H}/N{\left(A\right)}^{1H}}{I{\left(\mathrm{IR}\right)}_{i}^{1H}/N{\left(\mathrm{IR}\right)}^{1H}}\right)/\left(\frac{I{\left(Z\right)}_{i}^{X}/N{\left(\text{Z}\right)}^{X}}{I{\left(\mathrm{IR}\right)}_{i}^{X}/N{\left(\mathrm{IR}\right)}^{X}}\right)$$

***Note:*** where A stands for the solvent; *n*(Z)_i_ represents the content of substance Z (here is the salt) in the electrolyte after i cycles or the titrant; *I*(Z) is the integral area of the ^1^H or ^19^F spectrum of substance Z; X represents the characteristic nucleus of fluorine or hydrogen containing additive C.

23. Calculate the absolute content of different electrolyte components using the equation [5 min]:
  $$\;n{\left(Z\right)}_{i}=\left(\frac{I{\left(Z\right)}_{i}^{X}/N{\left(Z\right)}^{X}}{I{\left(\mathrm{IR}\right)}_{i}^{X}/N{\left(\mathrm{IR}\right)}^{X}}\right)/\left(\frac{I{\left(D\right)}_{i}^{1H}/I{\left(\mathrm{IR}\right)}_{i}^{1H}}{I{\left(D\right)}_{\text{blank}}^{1H}/I{\left(\mathrm{IR}\right)}_{\text{blank}}^{1H}}\right)\times \frac{{\text{m}}_{3}}{{\text{M}}_{\text{IR}}}\times \frac{{\text{V}}_{1}}{{\text{V}}_{2}}$$
  ***Note:*** Here, D represents the deuterated solvent; V_1_ represents the volume of diluent and V_2_ is the volume of the extracted solution; m_3_ is the mass of the internal standard and M_IR_ is the molar mass of the internal standard. In this protocol, $N{\left(\mathrm{DMC}\right)}^{1H}$ is 6, $N{\left(\mathrm{DCBF}\right)}^{1H}$ is 3, $N{\left(\mathrm{FSI}\right)}^{19F}$ is 2, $N{\left(\mathrm{MA}\right)}^{1H}$ is 2, $N{\left(\mathrm{DCBF}\right)}^{19F}$ is 3, $m_{3}$ is 32.9 μg, $M_{\mathrm{IR}}$ is 215.00 g mol^−1^, and V_2_ is 0.60 mL. The function of $\frac{I{\left(D\right)}_{i}^{1H}/I{\left(\mathrm{IR}\right)}_{i}^{1H}}{I{\left(D\right)}_{\text{blank}}^{1H}/I{\left(\mathrm{IR}\right)}_{\text{blank}}^{1H}}$ is to cross-calibrate the integration between different samples. An example of the calculated results can be found in Table 2, where the “integral area” is the raw integral data and the “calibrated area” represents the data after applying the cross-calibration function. Calculate the absolute loss of different electrolyte components by Δn =$n{\left(Z\right)}_{i}-\;n{\left(Z\right)}_{0}$.
  a. Calculate the irreversible capacity caused by inactive Li0, using $irre.capacity=\Delta n{\left(\mathrm{titrant}\right)}_{i}\times F$, where $\Delta n_{i}$ stands for the difference between the 0 cycle and the i cycle and F stands for the Faraday constant in mAh mol^−1^.
24. Repeat steps 3–25 to gather sufficient data [2 days].
  a. At least two sets of data from two different C-rates should be used.
25. Fit ETNs by multiple linear regression [30 min].
  a. Input the consumption of solvent, the consumption of anion, and the irreversible capacity from electrolyte decomposition into the MATLAB curve fitting tool as X, Y, and Z data.
  b. Perform the multiple linear regression with a custom equation Z=(z_1_X+z_2_Y)⋅F (where the faraday constant F=26.8 mAh mol^−1^) to fit the plot with the default least-squares algorithm.

***Note:*** The choice of the fitting equation is based on the observation that both capacity decay and the consumption of electrolyte exhibits linear behavior at the test condition. This particular protocol only deals with the simple case with linear capacity decay and linear electrolyte consumption behavior. Nonlinear behavior is beyond the scope of this protocol.

## Expected outcomes

**Figure 5:**
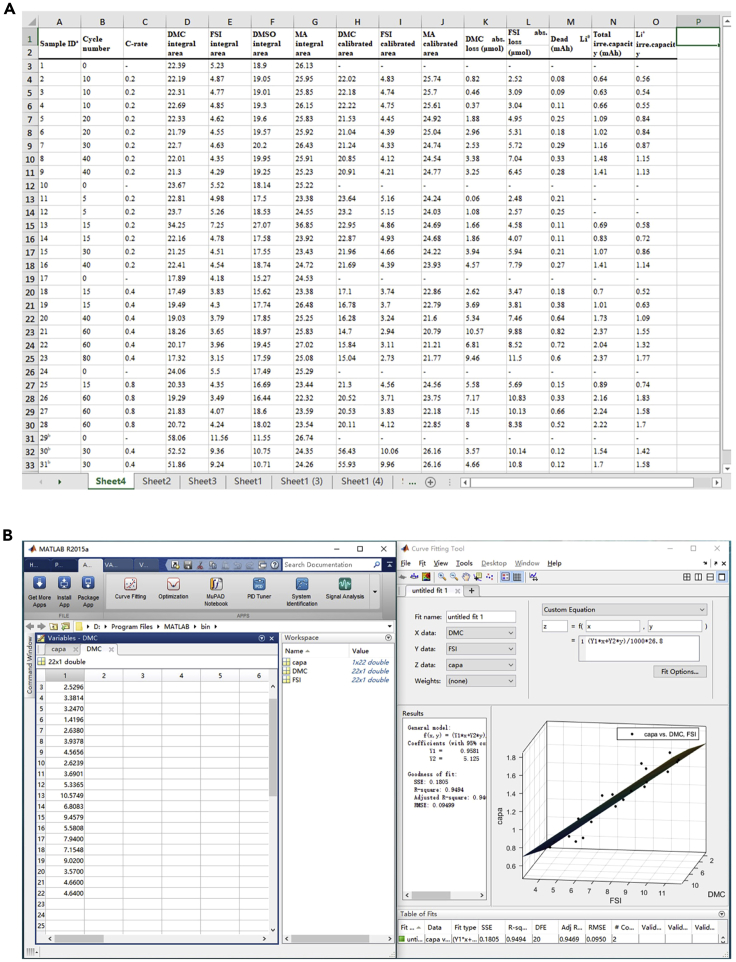
Examples for date processing and expected outcomes (A) Calculated qNMR results using Excel. The input results were partly reproduced with permission from Zhou et al.^1^ (B) Multiple linear regression fitting of ETNs using MATLAB.

By successfully employing the protocol, the evolution of the electrolyte and the accumulation of dead lithium can be evaluated accurately (as shown in Figure 5) with a ∼8 times reduced NMR testing time and a 70% reduced standard deviation, compared with the procedure without the use of relaxation enhancer, titrant, and cross-calibration.^1^ This will help to quickly determine the average decomposition rates of different electrolyte components without losing accuracy. For instance, based on the results from Zhou et al.,^1^ the root mean square error (RMSE) of the linear fitting for solvent and anion are 0.42 μmol and 0.45 μmol, respectively, when the decomposition rates are 0.14 μmol per cycle of anions and 0.09 μmol per cycle of solvents. Furthermore, the ETNs of electrolyte decomposition reactions can be determined (1.0 for DMC and 5.1 for LiFSI) with the RMSE of 0.095 mAh for the multiple regression and standard error of 0.44 and 0.31 for solvent and anion, respectively.^1^

Given that the universality of this method is not limited by the type of electrolyte, this work can also inspire the study of other battery working conditions (e.g., at high and low temperatures and calendar aging conditions), other types of anodes that do not involve excessive lithium metal (e.g., graphite and silicon-based anode) and other electrolyte components (e.g., co-solvents and functional additives).

## Limitations

The main limitation of the protocol is the requirement of the anode-free, or specifically lithium-metal-free, configuration. Because of the limited titration capacity of the titrant, the batteries containing a large amount of lithium metal may affect the accuracy and reproducibility of the method. The slow but somehow unavoidable reactions between titrant and other components may also exaggerate the calculation of inactive lithium metal. Besides, as an ex-situ instrument-based experiment, the quality of the results depends both on the proficient skill of sample preparation and the configuration of the NMR spectrometer, which may be compensated by repeated tests. Lastly, The qNMR measurements are limited to nuclei with high gyromagnetic ratios and natural abundances, such as ^1^H and ^19^F. The quantitative experiments of nuclei such as ^15^N, ^17^O, and ^31^P are beyond the scope of this protocol and require further exploration.^11^

## Troubleshooting

### Problem 1

In the battery cycling step (step 2), the battery experience overcharging.

### Potential solution

The overcharging in this protocol is mainly caused by the trace of water in battery components because DMC used as the solvent in this protocol can tolerate a high voltage of 4.3 V. Further drying, like keeping the materials in the glove box for one week, would solve this problem.

### Problem 2

The batteries exhibit very inconsistent behavior in terms of the capacity decay curve (step 3).

### Potential solution

Both the electrolyte and copper foil can influence the capacity decay behavior of anode-free batteries. To ensure their consistency, it is suggested to use the same batch of electrolytes and clean the copper foil with ethanol before use. Noted that, although the measured results can be normalized by the measured capacity, the consistency of cathodes is critical for evaluating the consistency of results under coordinates of the number of cycles.

### Problem 3

After the sample preparation step (step 11), the extract in the NMR sample tube is turbid.

### Potential solution

The black suspended solids are the fragment of the cathode that dropped during shaking. Resting the NMR tube for a while can settle the sediment at the bottom of the tube, where it would not interfere with the NMR measurement.

### Problem 4

During qNMR measurement (step 17), the width of peaks broadens in cycled samples.

### Potential solution

The peak broadening is caused by the high concentration of the paramagnetic substance in samples, most likely from dissolved cobalt and manganese from the cathode. Control and decreasing the amount of relaxation enhancer (m_3_) can solve this problem.

### Problem 5

The qNMR results show abnormally small quantities of MA (step 23).

### Potential solution

The incomplete dissolution could cause this problem. Although the concentration of MA (0.1 M) has not reached the reported solubility of DMSO-d6 (∼0.2 M), the dissolution of MA is relatively slow. Shaking and mixing for a longer time or simply prolonging the dissolution time for 3 days can solve the problem.

### Problem 6

The qNMR results show abnormal quantities of all substances (step 23).

### Potential solution

Although theoretically the concentration of internal reference is known, the actual concentration of internal reference may deviate from the theoretical value due to uncontrolled factors during sample preparation steps, e.g., the internal reference may not be totally dissolved (sticks on the wall) and the volume of extract may not be the recorded value (contains insoluble sediments), or, simply due to weighing and transferring error. The cross-calibration by the in-built DMSO signal can solve this problem. For a detailed explanation, please refer to Zhou et al.^1^

## Resource availability

### Lead contact

Further information and requests for resources and reagents should be directed to and will be fulfilled by the lead contact, Qiang Zhang (zhang-qiang@mails.tsinghua.edu.cn).

### Materials availability

This study did not generate new unique reagents.

## Data Availability

This paper does not report original code. All data reported in this paper will be shared by the lead contact upon request.
